# Use of a digital phantom developed by QIBA for harmonizing SUVs obtained from the state-of-the-art SPECT/CT systems: a multicenter study

**DOI:** 10.1186/s13550-017-0300-5

**Published:** 2017-06-20

**Authors:** Tadaki Nakahara, Hiromitsu Daisaki, Yasushi Yamamoto, Takashi Iimori, Kazuyuki Miyagawa, Tomoya Okamoto, Yoshiki Owaki, Nobuhiro Yada, Koichi Sawada, Ryotaro Tokorodani, Masahiro Jinzaki

**Affiliations:** 1 Department of Radiology, Keio University School of Medicine, 35 Shinanomachi, Shinjuku-ku, Tokyo, 160-8582 Japan; 2 Department of Radiological Technology, Gunma Prefectural College of Health Sciences, 323-1 Kamioki-machi, Maebashi, Gunma 371-0052 Japan; 3 Department of Radiology, Shimane University Hospital, 89-1 Enya-cho, Izumo, Shimane 693-8501 Japan; 4 Department of Radiology, Chiba University Hospital, 1-8-1 Inohana, Chuo-ku, Chiba-shi, Chiba, 260-8677 Japan; 5 Department of Radiology, Kochi Medical Hospital, 2125-1 Ike, Kochi-shi, Kochi, 781-8555 Japan; 6 Department of Radiology, Tsugaru General Hospital, 12-3 Iwaki-machi, Goshogawara-shi, Aomori, 037-0074 Japan; 7 Department of Radiological Sciences, Tokyo Metropolitan University, 7-2-10 Higashiogu, Arakawa-ku, Tokyo, 116-8551 Japan

**Keywords:** SPECT/CT, Harmonization, SUV, Multicenter study

## Abstract

**Background:**

Although quantitative analysis using standardized uptake value (SUV) becomes realistic in clinical single-photon emission computed tomography/computed tomography (SPECT/CT) imaging, reconstruction parameter settings can deliver different quantitative results among different SPECT/CT systems. This study aims to propose a use of the digital reference object (DRO), which is a National Electrical Manufacturers Association (NEMA) phantom-like object developed by the Quantitative Imaging Biomarker Alliance (QIBA) fluorodeoxyglucose-positron emission tomography technical committee, for the purpose of harmonizing SUVs in Tc-99m SPECT/CT imaging.

**Methods:**

The NEMA body phantom with determined Tc-99m concentration was scanned with the four state-of-the-art SPECT/CT systems. SPECT data were reconstructed using different numbers of the product of subset and iteration numbers (*SI*) and the width of 3D Gaussian filter (3DGF). The mean (SUV_mean_), maximal (SUV_max_), and peak (SUV_peak_) SUVs for six hot spheres (10, 13, 17, 22, 28, and 37 mm) were measured after converting SPECT count into SUV using Becquerel calibration factor. DRO smoothed by 3DGF with a FWHM of 17 mm (DRO_17 mm_) was generated, and the corresponding SUVs were measured. The reconstruction condition to yield the lowest root mean square error (RMSE) of SUV_means_ for all the spheres between DRO_17 mm_ and actual phantom images was determined as the harmonized condition for each SPECT/CT scanner. Then, inter-scanner variability in all quantitative metrics was measured before (i.e., according to the manufacturers’ recommendation or the policies of their own departments) and after harmonization.

**Results:**

RMSE was lowest in the following reconstruction conditions: *SI* of 100 and 3DGF of 13 mm for Brightview XCT, *SI* of 160 and 3DGF of 3 pixels for Discovery NM/CT, *SI* of 60 and 3DGF of 2 pixels for Infinia, and *SI* of 140 and 3DGF of 15 mm for Symbia. In pre-harmonized conditions, coefficient of variations (COVs) among the SPECT/CT systems were greater than 10% for all quantitative metrics in three of the spheres, SUV_max_ and SUV_mean_, in one of the spheres. In contrast, all metrics except SUV_max_ in the 17-mm sphere yielded less than 10% of COVs after harmonization.

**Conclusions:**

Our proposed method clearly reduced inter-scanner variability in SUVs. A digital phantom developed by QIBA would be useful for harmonizing SUVs in multicenter trials using SPECT/CT.

## Background

Although physical quality of single-photon emission computed tomography/computed tomography (SPECT/CT) images such as image resolution and noise is worse than that of PET/CT images, recent studies suggested the possibility for the clinical application of quantitative SPECT/CT [1–3]. In 2010, Zeintl et al. reported that the advanced SPECT/CT technology facilitated quantitative Tc-99m SPECT imaging with excellent accuracy in both the phantom (error < 3.6%) and patient studies (error < 1.1%) [3]. In 2012, Seret et al. investigated the performance of the four state-of-the-art SPECT/CT systems (Philips Brightview XCT, General Electric Discovery NM/CT 670 and Infinia Hawkeye 4, and Siemens Symbia T6) in quantitative assessment using three-dimensional iterative reconstruction (3D-OSEM) with attenuation and scatter corrections and resolution recovery [1]. Quantitative errors of the four SPECT/CT systems were less than 10% if the targets were several times larger than the spatial resolution of these SPECT devices. In the same year, Hughes et al. also conducted a phantom study in order to compare the images obtained with three different SPECT/CT systems [2]. Interestingly, their study showed no significant differences in image quality when using their own algorithm, whereas image quality was different between images reconstructed with the vendors’ reconstruction software. These results seem to raise a problem with regard to the standardization of SPECT/CT quantitation among different nuclear medicine institutions.

At present, common parameters used for quantitation in clinical SPECT/CT are the maximal standardized uptake value (SUV_max_) [4, 5] and peak SUV (SUV_peak_) [6]. SUV is the ratio of the radioactivity concentration in a voxel of the target to the average radioactivity concentration in the body, and SUV_max_ is the highest SUV within a volume of interest (VOI). Although SUV_max_ is preferably used in clinical PET imaging because it is not affected by ROI settings, optimization of reconstruction parameter settings is important to harmonize quantitative metrics among different PET cameras [7]. Since SUV is susceptible to spatial resolution and image noise, reconstruction conditions should be properly adjusted for each camera to provide reliable and robust SUVs in terms of the harmonization of SPECT/CT quantitation. In other words, harmonization-specific imaging protocol is crucial for clinical multicenter trials using quantitative SPECT/CT. This trend has been preceded by fluorodeoxyglucose-positron emission tomography/computed tomography (FDG-PET/CT) for multicenter trials [7, 8].

Recently, the Quantitative Imaging Biomarker Alliance (QIBA) FDG-PET technical committee has developed an FDG-PET/CT digital reference object (DRO) that is a synthetic test object representing an FDG-PET image volume in the Digital Imaging and Communications in Medicine (DICOM) format [9]. The DRO images in both PET and CT are based on the body phantom of National Electrical Manufacturers Association (NEMA) and International Electrotechnical Commission (IEC) [10]. Since the DRO is created synthetically with no random image noise, the DRO can be used as a reference standard to test SUV calculations. Pierce et al. used the DRO, which was smoothed by partial voxel computation in view of finite spatial resolution, to ensure the standardization of SUV computation in PET between medical image viewing workstations [11]. According to the Japanese guideline for oncological FDG-PET/CT imaging in 2009, a DRO-like digital phantom smoothed by a 3D Gaussian filter (3DGF) with a FWHM of 10 mm was used as a reference in order to define prerequisite image quality for detection of a 10-mm hot sphere with SUV of 4 [12].

In the present study, we propose a use of the DRO smoothed by 3DGF with a FWHM of 17 mm (DRO_17 mm_) for the purpose of harmonizing SUVs in Tc-99m SPECT/CT imaging. Our phantom study using the aforementioned four state-of-the-art SPECT/CT systems to image NEMA phantom showed that a 10-mm hot sphere was undetected and a 13-mm hot sphere was barely discernible, whereas all the scanners clearly depicted a 17-mm sphere. Based on the detectable feature, we hypothesized that DRO_17 mm_ could be used as a reference to determine the harmonization-specific imaging protocol as a digital phantom with a smooth of 10 mm which was used in the Japanese PET guideline [12]. The aim of this study was to demonstrate the feasibility of SUV harmonization among these SPECT/CT using DRO_17 mm_ as a reference standard.

## Methods

### Determination of Tc-99m concentration in NEMA phantom to simulate clinical Tc-99m SPECT/CT

**Fig. 1:**
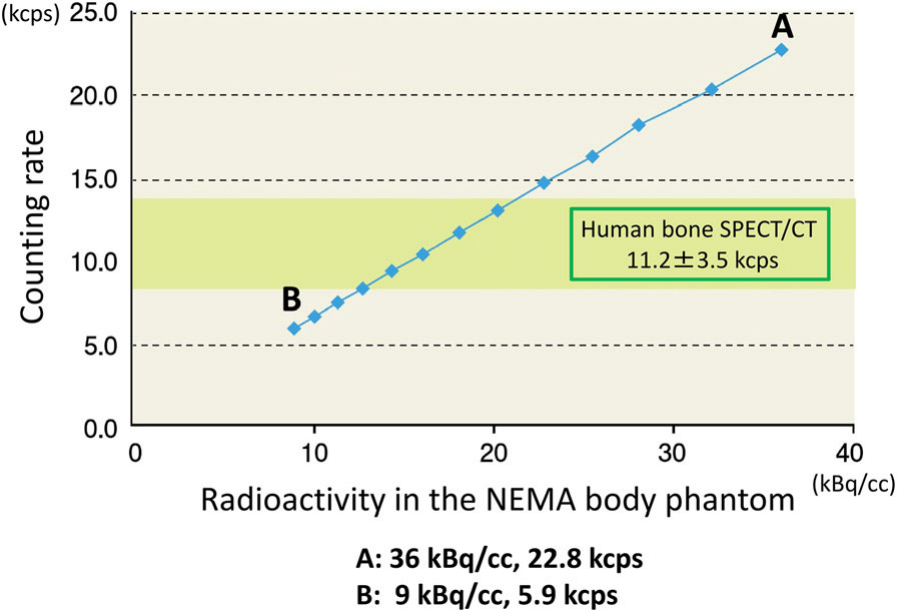
Correlation of Tc-99m concentration of the NEMA phantom and counting rate in Discovery NM/CT 670 from the beginning (*A*) to the end (*B*) of the SPECT scan. There was a linear relationship between the radioactivity and counting rate. Tc-99m concentration for further phantom study was determined based on the counting rate of clinical bone SPECT/CT

In order to determine Tc-99m concentration enclosed in the NEMA body phantom, the following procedure was performed; first, SPECT/CT scans using an integrated SPECT/CT system (Discovery NM/CT 670pro, GE Healthcare) equipped with a low-energy high-resolution collimator were performed in 28 cancer patients 3 h after intravenous injection of 740 MBq of Tc-99m hydroxymethylene diphosphonate (Tc-99m HMDP) at one of the institutions participating in the present study. The SPECT data obtained from routine clinical examinations were used in order to determine Tc-99m concentration in the NEMA phantom, which was approved by the Institutional Review Board (IRB) in the hospital. The IRB officially granted permission for this retrospective review of the imaging data and waived the need for obtaining informed consent from the patients. SPECT counts of the lower abdominal portion were measured in order to obtain the reference counting rates (11.2 ± 3.5 kilo counts per second (kcps)) for the phantom study. Second, the body phantom in which six hot spheres (10, 13, 17, 22, 28, and 37 mm) were embedded was filled with Tc-99m solution so that the spheres had a 4:1 radioactivity ratio compared with the background. At the beginning of the SPECT scan, the radioactivity concentration and the SPECT counting rate of the phantom were 36 kBq/cc and 22.8 kcps, respectively (Fig. 1). Then, 6-min SPECT/CT scans were performed repeatedly with an interval of 60 min for 12 h. Based on the results of the correlation between the radioactivity concentration and the SPECT counting rate, the optimal radioactivity concentration for further phantom studies were determined.

### Calculation of calibration factor for SUV measurement using a cylindrical phantom

A cylindrical phantom with a diameter of 160 mm and a height of 150 mm (3016 mL) filled with Tc-99m solution of known activity concentration (approximately 25 MBq) was scanned for 6 min. Data were reconstructed with 3D-OSEM with scatter and CT-based attenuation correction and were processed with various parameter settings including the pre-harmonized conditions used in each of the four SPECT/CT cameras. Basic performances of the SPECT/CT cameras were describe elsewhere [1], and the detailed imaging conditions and collimator configurations are shown in Table 1. Parameter settings are comprised of the product of subset and iteration numbers (*SI*, range 40–160) and 3DGF (range 1.0–4.0 pixel (Infinia and Discovery; pixel size, 4.4 mm) or 5–17 mm (Brightview and Symbia)). The processing of 3DGF in Philips Brightview XCT was performed using a commercially available software GI-PET (AZE Co., Ltd., Tokyo, Japan) because this filter option was not installed in any imaging workstation belonging to the institution with Philips Brightview XCT. Resolution recovery (RR) by compensating the distance-dependent detector response was used.

**Table 1:** Imaging conditions and collimator configurations regarding the four state-of-the-art SPECT/CT systems

	Brightview XCT	Discovery NM/CT 670	Infinia Hawkeye 4	Symbia T6
Imaging condition				
Step and shoot image acquisition				
No. of step	30	30	30	30
Rotation angle	6	6	6	6
No. of projection	60	60	60	60
Scan orbit	body contour	body contour	body contour	body contour
Size for image acquisition				
Matrix (*x*, *y*, *z*)	132, 132, 132	128, 128, 128	128, 128, 128	128, 128, 128
Pixel size and slice thickness (mm)	4.7	4.4	4.4	4.8
Smoothing filter	3D Gaussian	3D Gaussian	3D Gaussian	3D Gaussian
Reconstructed image for ROI analysis				
Matrix (*x*, *y*, *z*)	256, 256, 203	256, 256, 207	256, 256, 130	256, 256, 187
Pixel size and slice thickness (mm)	2.0	2.0	2.2	2.0
Energy window				
Main	140.5 keV ± 10%	140.5 keV ± 10%	140 keV ± 10%	140 keV ± 10%
Sub	N.A.	120 keV ± 5%	120 keV ± 5%	120 keV ± 5%
Attenuation correction	CT-based	CT-based	CT-based	CT-based
Scatter correction	ESSE	DEW	DEW	DEW
Collimator				
Type	CHR	LEHR	LEHR	LEHR
No. of holes (thousand)	40.2	86.3	86.3	148
Hole shape	Hexagon	Hexagon	Hexagon	Hexagon
Hole length (mm)	48	35	35	24.1
Septal thickness (mm)	0.15	0.20	0.20	0.16
Hole diameter across the flats (mm)	2.03	1.50	1.50	1.11

*ESSE* effective source scatter estimation method. *DEW* dual-energy window method, *CHR* cardiac high-resolution collimator, *LEHR* low-energy high-resolution collimator, *N.A.* not applicable

SPECT/CT data in each reconstruction condition were analyzed using a commercially available software GI-BONE (AZE Co., Ltd., Tokyo, Japan). With the software, slice thickness was automatically converted to be about 2 mm to allow isotropic voxel evaluation (Table 1). A circular ROI was drawn on the center of the cylindrical phantom in the central slice as well as in slices ±1 and ±2 cm away, measuring SPECT count density (count/cc). The calibration factor was calculated as the ratio of actual radioactivity concentration (as measured by the dose calibrator) in the phantom at the time of scanning (ACC) to the measured SPECT count density per scan duration (MC), and we call this factor Becquerel calibration factor (BCF). Consequently, the BCF is calculated as:

$$ {\mathrm{BCF}}_{\left[\mathrm{Bq}/\mathrm{cps}\right]}=\frac{{\mathrm{ACC}}_{\left[\mathrm{Bq}/\mathrm{cc}\right]}}{{\mathrm{MC}}_{\left[\mathrm{count}/\mathrm{cc} \times 1/ \sec \right]}} $$

The BCF should be dependent on the performance of SPECT/CT system and imaging conditions. The MC also should be affected by a scaling factor (multiplying pixel count in reconstruction with RR) in GE resolution modeling.

### SUV conversion of NEMA body phantom image using BCF

In order to simulate clinical Tc-99m SPECT/CT scans, the activity concentration levels in the background and spheres in the NEMA body phantom were set at 18 and 54 kBq/cc, respectively (Fig. 1). The phantom was scanned for 6 min with the four different SPECT/CT systems. The phantom images were reconstructed in the same parameter setting as BCF images. The phantom data in each reconstruction condition were analyzed using the same software as BCF data. Six different target ROIs, whose diameters were equal to the physical inner diameters of the hot spheres, were placed on the target slice. The *SUV* is calculated as:

$$ \mathrm{S}\mathrm{U}\mathrm{V}={\mathrm{BCF}}_{\left[\mathrm{Bq}/\mathrm{cps}\right]}\times {\mathrm{MC}}_{\left[\mathrm{count}/\mathrm{cc}\kern0.5em \mathrm{g}\kern0.5em 1/ \sec \right]}\times \frac{{\mathrm{Body}\ \mathrm{weight}}_{\left[\mathrm{g}\right]}}{{\mathrm{Injected}\ \mathrm{activity}}_{\left[\mathrm{Bq}\right]}} $$

In this phantom study, the reciprocal of body weight per injected dose was 9000 Bq/g so that background SUV was 1. Regarding calculation of SUVs, 10-, 13-, 17-, 22-, 28-, and 37-mm circular ROIs were drawn exactly on the corresponding spheres in the central slice by following the CT boundaries of the fused SPECT/CT images. Then, SUV_peak_, SUV_max_, and the mean SUV (SUV_mean_) for the spheres were measured. Peak *SUV* represents the average *SUV* obtained within a 1-cc sphere of region of interest (ROI) centered on a highest voxel of the target area.

### Harmonization of SUVs using DRO_17 mm_

**Fig. 2:**
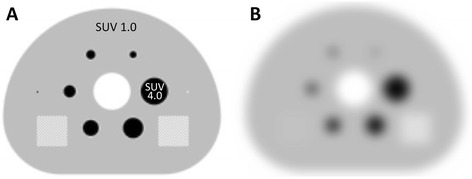
**a** Phantom configuration of a digital reference object (DRO) and **b** DRO filtered by a 17-mm Gaussian filter (DRO_17 mm_). The *square boxes* in DRO are by design (not used in the present study)

Simulated images of original DRO and DRO_17 mm_ are shown in Fig. 2. SUV_mean_, SUV_peak_, and SUV_max_ of the six spheres in DRO_17 mm_ are described in Table 2. As a measure of harmonization of reconstruction conditions, the root mean square error (RMSE) was measured; RMSE is the square root of the variance in SUV_mean_ of the six spheres between DRO_17 mm_ and actual phantom images obtained with the SPECT/CT cameras. Therefore, RMSE is measured as:

**Table 2:** SUV values derived from a digital reference object smoothed by a 17-mm Gaussian filter (DRO_17 mm_)

	Quantitative metrics		
	Quantitative metrics		
	SUV_mean_	SUV_peak_	SUV_max_
Sphere diameter (mm)			
10	1.18	1.17	1.20
13	1.36	1.38	1.42
17	1.67	1.77	1.86
22	2.12	2.40	2.53
28	2.55	3.14	3.29
37	2.94	3.82	3.91

$$ \mathrm{RMSE}=\sqrt{\frac{1}{6}{\displaystyle \sum_{i=10,13,17,22,28,37\ \mathrm{mm}}}{\left({\mathrm{SUV}}_{\mathrm{mean}\ \mathrm{i}\mathrm{n}\ \mathrm{phantom},\  i}-{\mathrm{SUV}}_{\mathrm{mean}\ \mathrm{i}\mathrm{n}\ \mathrm{DRO}17\mathrm{mm},\ \mathrm{i}}\right)}^2} $$

Reconstruction conditions according to the manufacturers’ recommendation or the policies of their own departments are shown in Table 3. SUV_mean_ of the hot spheres and RMSE in the pre-harmonized conditions are also shown in Table 3.

**Table 3:** Reconstruction conditions according to the manufacturers’ recommendation or the policies of their own departments

	SPECT/CT scanner			
	BrightView	Discovery	Infinia	Symbia
Reconstruction parameter				
Subset	8	10	10	10
Iteration	10	10	10	10
Filter	Gaussian	Gaussian	Gaussian	Gaussian
Cutoff value	15 mm	2.5 pixel	2.5 pixel	9 mm
Resolution recovery	Astonish	Evolution	Evolution	FLASH 3D
SUV_mean_ of the spheres				
10 mm	1.08	1.25	0.98	1.06
13 mm	1.14	1.48	1.28	1.37
17 mm	1.38	1.73	1.73	1.99
22 mm	1.81	2.19	1.92	2.52
28 mm	2.41	2.96	2.42	3.08
37 mm	2.86	3.21	2.71	3.50
RMSE	0.20	0.20	0.13	0.40

RMSE was calculated in the following conditions: *SI*, range 40–140; 3DGF, range 1.0–4.0 pixel (Infinia and Discovery) or 5–17 mm (Brightview and Symbia). Then, for every scanner examined, settings were found that showed a clear optimum for harmonization. In both pre- and post-harmonized conditions, coefficient of variation (COV) of SUV_mean_, SUV_max_, and SUV_peak_ between the four scanners were calculated.

## Results

### Tc-99m concentration for phantom study

As shown in Fig. 1, Tc-99m concentration in the NEMA body phantom had linear correlation with the acquisition counting rate. The counting rates in human bone SPECT/CT (11.2 ± 3.5 kcps) were equivalent to Tc-99m concentration of 12.8–22.9 kBq/ml. Therefore, the activity concentration levels in the background and spheres for further evaluation were set at 18 and 54 kBq/cc, resulting in mean activity concentration of the entire phantom of approximately 18.3 kBq/cc.

### BCF measurement

Table 4 shows the distribution of BCF among SPECT/CT systems with different reconstruction conditions. The difference in BCF value was small between BrightView and Symbia and between Infinia and Discovery. A scaling factor seemed to affect the BCF. Reconstruction conditions did not significantly affect the BCF (approximately less than 3% of mean value).

**Table 4:** Distribution of BCF among SPECT/CT systems with different reconstruction conditions

	SPECT/CT scanner			
	BrightView	Discovery^a^	Infinia^a^	Symbia
Mean	5309	1617	1538	4914
Standard deviation	35	5	48	32
Relative standard deviation (%)	0.7	0.3	3.1	0.7

^a^A scaling factor is involved with the values

### Effects of reconstruction parameter settings on SUVs and RMSE

**Fig. 3:**
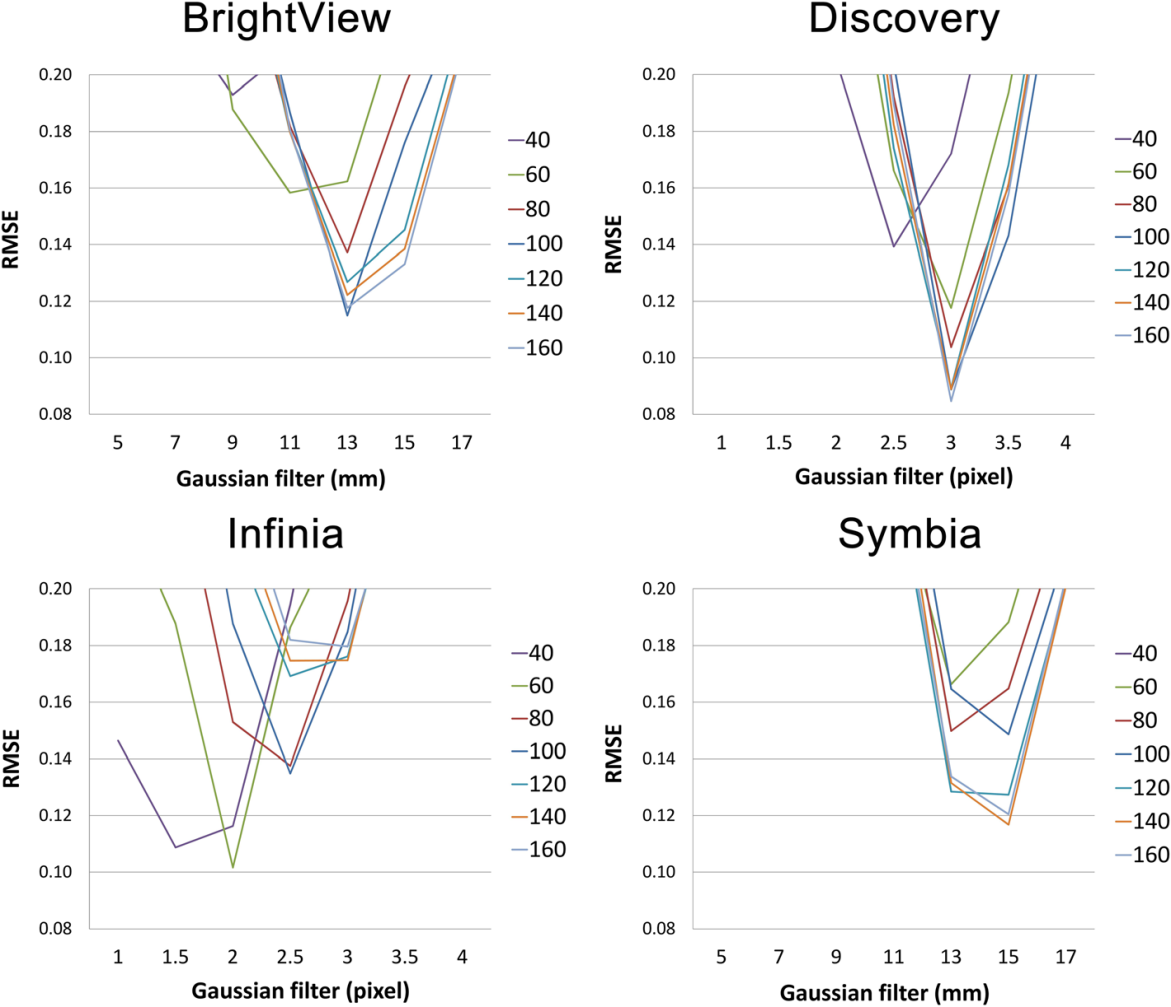
The root mean square error (RMSE) in SUV_mean_ of the six spheres between DRO_17 mm_ and actual phantom images obtained with the SPECT/CT cameras

Figure 3 shows RMSE for the four SPECT/CT systems. RMSE was lowest in the following reconstruction conditions: *SI* of 100 and 3DGF of 13 mm for Brightview XCT (RMSE = 0.115); *SI* of 160 and 3DGF of 3 pixels for Discovery NM/CT (RMSE = 0.085); *SI* of 60 and 3DGF of 2 pixels for Infinia (RMSE = 0.102); and *SI* of 140 and 3DGF of 15 mm for Symbia (RMSE = 0.117). It should be noted that the minimum RMSE was below 0.12 for each harmonized setting.

### SUV_mean_, SUV_max_, and SUV_peak_ in both pre- and post-harmonized conditions

**Fig. 4:**
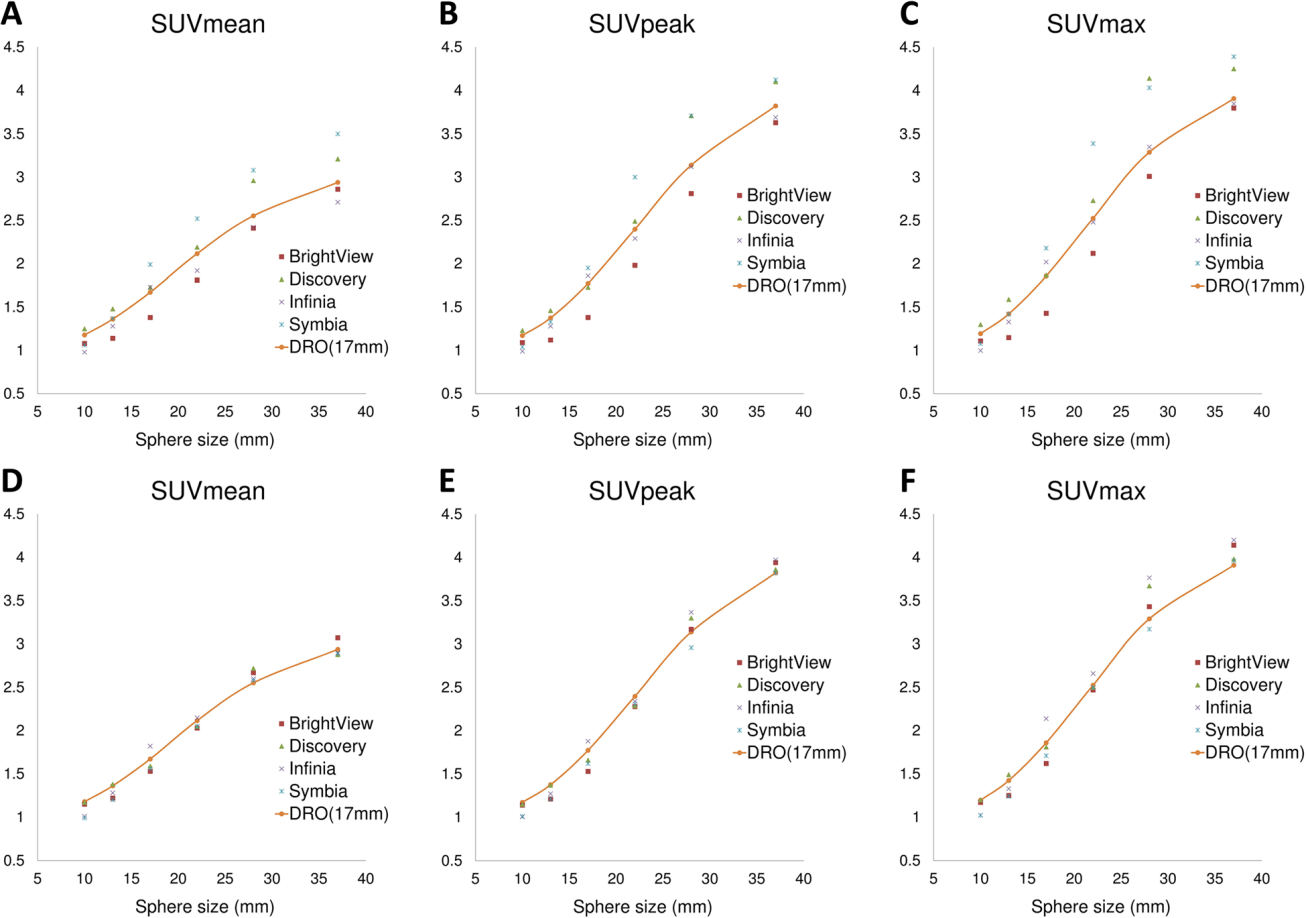
Inter-scanner variability in standardized uptake values (SUVs) **a**–**c** before and **d**–**f** after harmonization

Figure 4 shows SUV_mean_, SUV_max_, and SUV_peak_ of the hot spheres in both pre- and post-harmonized conditions. Table 5 shows COVs of these metrics between the four SPECT/CT systems. In pre-harmonized conditions, COVs were greater than 10% for all metrics in the 17-, 22-, and 28-mm spheres, SUV_max_ in the 13-mm sphere and SUV_mean_ in the 37-mm sphere. In contrast, all metrics except SUV_max_ in the 17-mm sphere yielded less than 10% of COVs after harmonization.

**Table 5:** Coefficient of variations (COVs) of SUVs between the four SPECT/CT systems

	Pre-harmonization			Post-harmonization		
Spheres	SUV_mean_	SUV_peak_	SUV_max_	SUV_mean_	SUV_peak_	SUV_max_
10 mm	9.00	8.23	9.81	7.47	6.42	7.48
13 mm	9.46	9.38	11.57	5.51	5.17	7.54
17 mm	12.70	12.52	14.90	7.05	7.66	10.77
22 mm	12.99	15.20	17.30	2.24	1.00	2.94
28 mm	11.24	11.63	12.93	2.11	4.85	6.56
37 mm	10.02	5.82	6.21	2.67	1.55	2.51

## Discussion

Recent advances in SPECT/CT technologies allowed major manufacturers to mass-produce commercial SPECT/CT systems for clinical application of not only SPECT/CT fusion imaging but also fully quantitative SPECT imaging. Although PET/CT has become an important diagnostic tool quantifying tracer uptake, only a small number of PET tracers have yet been approved in clinical practice. In contrast, there have already been various kinds of available radiopharmaceuticals labeled with single-photon emitters; much focus is being placed on the value of quantitative SPECT/CT [4, 5, 13–16]. Especially, clinical application of quantitative SPECT/CT using bone-seeking radiotracers is highly expected as shown in a successful report on the use of F-18 fluorine PET for prognostic assessment [17] as well as the accumulated evidences of quantitative planar bone scintigraphy in prostate cancer [18]. The bone scan index (BSI) [19, 20], which quantifies the total bone metastatic burden relative to the total skeletal mass on two-dimensional images, is getting wider acceptance as a biomarker for predicting survival in patients with prostate cancer [21–26]. However, there are substantial false-positive and false-negative findings when evaluating bone metastasis without SPECT/CT [27–31]. In addition, quantifying tracer accumulation on a per-lesion basis is limited by the projection of several overlying structures in a planar image. For instance, uptake in the sternum may contain some amounts of uptake in the thoracic spine in an anterior view of planar image, and quantitative analysis would therefore be difficult especially when metastasis occurs in these bones. We envisaged that harmonizing SUVs using the DRO could be applied to multicenter clinical trials using Tc-99m SPECT/CT; in particular, harmonized SUVs in bone SPECT/CT may become an alternative choice to BSI. In addition, the harmonization method might be utilized to reduce inter-scanner variability in measurement of SPECT/CT-derived absorbed doses in a variety of “theranostics” situations such as Tc-99m MAA SPECT/CT in Y-90 microsphere therapy, I-131 SPECT/CT in thyroid cancer therapy, and Lu-177-PSMA in prostate cancer therapy [32–34].

We used DRO for harmonization instead of a two-step approach of assessing how close each scanner can get to true SUV (i.e., SUV = 4) and then harmonizing to the lower common denominator based on the following reasons:

1. We found that both SUV_max_ and SUV_peak_ fluctuated when acquisition time or phantom radioactivity was changed, probably due to image noise. In contrast, SUV_mean_ did not (data not shown). Therefore, we thought that SUV_max_ and SUV_peak_ are not suitable parameters for harmonization in terms of test-retest reproducibility.
2. Although SUV_mean_ may be used for harmonization because of being unsusceptible to image noise, it never reached the uptake value of 4 even for the largest sphere (37 mm) due to partial volume effect. Hence, it seems impractical to assess how close each scanner can reach SUV_mean_ of 4. Instead, DRO was smoothed to match SUVs of the targets in each of SPECT/CT systems with the corresponding SUVs in DRO.
3. Presetting DRO as a reference allows a variety of institutions to easily join the harmonization projects without any revisions of previously harmonized protocols in enrolled institutions, whereas the two-step approach seems complex when many scanners need to be harmonized.

It is important to know how accurate the current SPECT/CT technologies can be in terms of quantitation. It goes without saying, however, that even the state-of-the-art SPECT/CT systems are less reliable than general PET/CT systems especially in quantifying small lesions due to limited detector sensitivity and intrinsic spatial resolution; not surprisingly, a 10-mm hot sphere was undetected with any of the four SPECT/CT systems under all reconstruction conditions, and a 13-mm hot sphere was barely discernible in most of the reconstructed SPECT images (data not shown). Based on the fact that point spread function or line spread function of the SPECT detectors, which represents image blurring due to finite spatial resolution, can be geometrically approximated by Gaussian function; DRO_10 mm_ or DRO_13 mm_ was considered unsuitable to serve as a reference image. In contrast, the actual hot spheres measuring at least 17 mm were clearly observed irrespective of reconstruction conditions and SPECT/CT systems. Therefore, DRO_17 mm_ was chosen as a reference image in the present study.

3DGF was used throughout the harmonization instead of using another filter such as Butterworth and Hanning filters. This is not only because DRO_17 mm_ was generated with Gaussian filter, but because measurement of BCF with Gaussian filter was more stable than that with Butterworth or Hanning filter when changing reconstruction parameters such as *SI* and cutoff value of these filters (data not shown).

We found that the lowest RMSE value was obtained with 3DGF of 8.8 to 15 mm and *SI* of 60 or 160 (Fig. 3). The results were partially different from the European Association of Nuclear Medicine (EANM) practice guidelines and recommendations of the camera manufacturers [35], which indicates a need for harmonization-specific imaging protocol. As shown in Fig. 4 and Table 5, inter-scanner variability in SUVs among the state-of-the-art SPECT/CT systems was clearly decreased after the proposed harmonization procedure. In this context, we propose the use of a digital phantom developed by QIBA for harmonizing SUVs in multicenter trials.

In PET/CT, the EANM guidelines do not positively recommend the use of resolution recovery for quantitative assessment in multicenter studies due to Gibbs artifact [36]. We observed small amount of uptake biased to a peripheral side of the sphere in the larger spheres (e.g., 37-mm sphere), probably due to Gibbs phenomenon. This effect possibly resulted in a slight elevation of SUV_max_ over true value (i.e., SUV = 4) as shown in Fig. 4. Although resolution recovery was responsible for the overshoot, the lack of resolution recovery significantly underestimated SUVs. For instance, SUV_max_ and SUV_mean_ of a 37-mm sphere without resolution recovery were about 3.3 and 2.5, respectively. In light of the principle of photon detection with collimator-dependent SPECT systems, it is reasonable to compensate the distance-dependent detector response for lesion-based quantitative assessment. At present, we consider that resolution recovery should be used at the sacrifice of the small amount of the overshoot.

In the present study, the radioactivity of the NEMA phantom was determined on the basis of the bone SPECT data under a 6-min acquisition protocol. According to the Japanese technological guidelines on nuclear imaging, bone SPECT data should be collected for 5–6 min/bed [37]. On the other hand, the EANM practice guidelines indicate the acquisition time of 10–30 min/bed [35]. The difference may be due to the fact that radioactive dose administered to patients undergoing bone scintigraphy is different between Japan and Europe (mean dose, 740 vs 500 MBq). In addition, considering the difference in body weight and height between Japanese (light and short) and European people (heavy and tall), 6 min SPECT acquisition for Japanese patients would be equivalent to 10 min or more acquisition for European patients in terms of SPECT counts per bed position.

There are several limitations in this study. First, DRO_17 mm_ has no absolute and universal significance as a reference image. It does not seem to be necessary to smooth to a level which matches the sphere size. In other words, DRO_14 mm_, DRO_15 mm_, or DRO_16 mm_ might serve as better references. In our preliminary study, DRO_17 mm_ was arbitrarily determined as a reference. However, it is worth noting that the minimum RMSE was below 0.12 for each harmonized setting (Fig. 3) and that the harmonized SUV curves as a function of sphere size are close to the curve of DRO_17 mm_ as shown in Fig. 4. Hence, we considered that DRO_17 mm_ could be a suboptimal reference for a multicenter study and that DRO_14 mm_, DRO_15 mm_, and DRO_16 mm_ may also be references for another multicenter study. In any case, it seems important to specify which DRO is regarded as a reference together with RMSE for each scanner. Our results suggest that RMSE of 0.12 may serve as an index of appropriateness of harmonization. Second, background and cold regions were not focused on in this study. This study was intended for a variety of multicenter SPECT studies such as bone SPECT/CT in which quantitation of background or cold regions would be unnecessary. In other words, the results of our study should not be applied to myocardial or cerebral perfusion SPECT/CT in which decrease in tracer uptake has significant impact on treatment strategy. In this context, we believe that our study is the first step to expand the use of DRO by QIBA for the future of quantitation using SPECT/CT. Finally, the currently available DRO has a contrast of 4:1. Therefore, we collected SPECT data of the NEMA phantom with 4:1 concentration ratio. Whether the results would be applicable for other contrast remains unknown. Examining this issue is one of the top priorities for further research.

## Conclusions

In the present study, the DRO smoothed by 3DGF with a FWHM of 17 mm was used for the purpose of harmonizing SUVs in Tc-99m SPECT/CT imaging. SUVs generated according to the manufacturers’ recommendation or the policies of their own departments had substantial inter-scanner variability, indicating a need for harmonization-specific imaging protocols. Our harmonization clearly reduced inter-scanner variability in all metrics except SUV_max_ in the 17-mm sphere with less than 10% of COVs. A digital phantom developed by QIBA would be useful for harmonizing SUVs in multicenter trials.

## Abbreviations

3DGF: Three-dimensional Gaussian filter
BCF: Becquerel calibration factor
BSI: Bone scan index
CHR: Cardiac high-resolution collimator
COV: Coefficient of variation
DEW: Dual-energy window method
DICOM: Digital imaging and communications in medicine
DRO: Digital reference object
ESSE: Effective source scatter estimation method
FDG: Fluorodeoxyglucose
HMDP: Hydroxymethylene diphosphonate
IEC: International Electrotechnical Commission
LEHR: Low-energy high-resolution collimator
NEMA: National Electrical Manufacturers Association
PET/CT: Positron emission tomography/computed tomography
QIBA: Quantitative imaging biomarker alliance
RMSE: Root mean square error
ROI: Region of interest
SPECT/CT: Single-photon emission computed tomography/computed tomography
SUV: Standardized uptake value
VOI: Volume of interest
